# The Role of Cytoplasmic mRNA Cap-Binding Protein Complexes in *Trypanosoma brucei* and Other Trypanosomatids

**DOI:** 10.3390/pathogens6040055

**Published:** 2017-10-27

**Authors:** Eden R. Freire, Nancy R. Sturm, David A. Campbell, Osvaldo P. de Melo Neto

**Affiliations:** 1 Department of Microbiology, Instituto Aggeu Magalhães, Fiocruz, Recife 50740-465, PE, Brazil; freireer@hotmail.com; 2 Department of Microbiology, Immunology & Molecular Genetics, David Geffen School of Medicine, University of California at Los Angeles, Los Angeles, CA 90095, USA; nsturm.ucla@gmail.com (N.R.S.); dc@ucla.edu (D.A.C.)

**Keywords:** Kinetoplastids, eIF4E, translation initiation

## Abstract

Trypanosomatid protozoa are unusual eukaryotes that are well known for having unusual ways of controlling their gene expression. The lack of a refined mode of transcriptional control in these organisms is compensated by several post-transcriptional control mechanisms, such as control of mRNA turnover and selection of mRNA for translation, that may modulate protein synthesis in response to several environmental conditions found in different hosts. In other eukaryotes, selection of mRNA for translation is mediated by the complex eIF4F, a heterotrimeric protein complex composed by the subunits eIF4E, eIF4G, and eIF4A, where the eIF4E binds to the 5′-cap structure of mature mRNAs. In this review, we present and discuss the characteristics of six trypanosomatid eIF4E homologs and their associated proteins that form multiple eIF4F complexes. The existence of multiple eIF4F complexes in trypanosomatids evokes exquisite mechanisms for differential mRNA recognition for translation.

## 1. Regulation of Gene Expression in *Trypanosoma brucei* and Related Trypanosomatids

*Trypanosoma brucei* and closely related organisms (collectively called trypanosomatids) are uniflagellated parasitic protozoa belonging to the eukaryotic lineage Excavata and classified within the order Kinetoplastida [1]. These organisms evolved from free-living phagotrophic protozoa (similar to their current sister group, the Bodonids) into well-adapted parasitic organisms, having undergone major changes that include serial loss of genes involved in macromolecular digestion and the assimilation and multiplication of membrane transporters for scavenging metabolites from the host [2,3,4]. The family Trypanosomatidae is composed, to date, of 19 genera of Monoxenous (one host, insect) and Dixenous (two hosts, insect and vertebrate or plant) parasites [5]. Two of those, *Trypanosoma* and *Leishmania*, are medically relevant, and have elicited substantial interest from the scientific community, due to their role in diseases that affect millions of people. These diseases are found mainly in tropical and subtropical areas of the world [6,7], and poignantly lack effective vaccines and means or proper control, with the currently available treatments based on the administration of toxic drugs [8].

A number of unique features rarely seen in other eukaryotes characterize the basic biology of trypanosomatids. An example is the peculiar way they control their gene expression, critical for their adaptation to parasitic life in different hosts [9]. Little regulation of transcription is observed, with their genes organized in clusters that are constantly transcribed into long polycistronic precursor mRNAs [10,11]. Genes in the same cluster are typically functionally unrelated [12], and transcription initiation regions lack the usual RNA Pol II promoters, unlike other eukaryotes, but are controlled epigenetically [13,14]. Following synthesis, the long transcripts are processed into mature mRNAs, having individual coding sequences, by another unusual process called *trans*-splicing [15]. During *trans*-splicing, the polycistronic precursor is cleaved, and a mini-exon spliced leader (SL) is added to each monocistronic unit, defining the 5′ terminus of the mRNA. The subsequent polyadenylation of its 3′ end, in a manner reminiscent of other eukaryotes but dependent on different *cis*-acting elements, defines the mature mRNA 3′ termini [16,17]. The SL *trans*-splicing process was first described in trypanosomatids [18,19], and then later found to be present in many other eukaryotes, including cnidarians, ctenophores, rotifers, platyhelminthes, nematodes, crustaceans, sponges, chaetognaths, tunicates, dinoflagellates, and euglenids [20]. The conserved, 39 nt long, trypanosomatid mini-exon is derived from a short precursor SL RNA transcript, a product of roughly 100–200 tandem SL RNA genes [21]. The SL mini-exon is characterized by a highly modified 5′-cap structure, consisting of the classical m^7^GTP (7-methylguanine) cap found at the 5′-end of eukaryotic mRNAs, followed by modifications of the first four transcribed nucleotides of the SL sequence. These nucleotides (AACU) are modified by the addition of 2′-*O*-methylations to all four ribose moieties, and by unusual base-methylations found on the first adenine (m_2_^6^A) and fourth uridine (m^3^U) [15,22]. The classical cap plus the four modified nucleotides constitute the so called cap4 structure, unique to trypanosomatids and involved in mRNA translation [23,24]. The m^7^GTP cap addition and modifications to the cap4 structure on the SL RNA occurs co-transcriptionally during the biogenesis of the SL precursor [25,26,27], and are necessary for its efficient use during *trans*-splicing [28,29]. After maturation through proper *trans*-splicing and polyadenylation, the protein coding mRNAs are exported to the cytoplasm, to be recognized by the translation machinery [30].

Although the lack of traditional control for the trypanosomatid polycistronic transcription suggests that adjacent genes would have the same levels of expression, it is known that, in fact, many show strikingly different steady-state levels [11]. Additionally, a large fraction of the proteomes of representative species exhibit changes according to their life stages, a likely consequence not only of changes in mRNA and protein stability [31], but also in translation efficiency rates, selectively targeting specific mRNAs that may play a significant role during the trypanosomatid complex life cycle [32]. Hence, it is believed that control of gene expression in trypanosomatids is based on mechanisms targeting post-transcriptional events, such as *trans*-splicing, mRNA transport, turnover, and translation [9]. The present review details the knowledge acquired recently on mRNA translation and its regulation in trypanosomatids through the study of the multiple homologues identified in these organisms of the mRNA cap binding protein, also known as eIF4E. First, however, a brief overview on the basics of the eukaryotic translation initiation and eIF4E is required.

## 2. The Eukaryotic Translation Initiation

Translation of mRNAs into proteins represents the ultimate goal of gene expression, essential for the survival of all living organisms. As one of the most conserved cellular processes, it is controlled by several protein factors that act upon four main stages: Initiation, Elongation, Termination, and Recycling [33]. Translation regulation can be mediated by mechanisms acting at any of these stages, but the main target is the Initiation stage, where a large number of accessory proteins, called eukaryotic initiation factors (eIFs), are required. The eIFs participate in many critical steps required for translation initiation to proceed, and these include the identification of mature transcripts, their protection against nucleases, and recruitment to the ribosomes for the start of translation properly [33].

In summary, translation initiation begins with the assembly of the 43S “pre-initiation complex” (PIC), composed of the Met-tRNAi, the tRNA carrying the anticodon for the first AUG, plus several initiation factors (eIF1, eIF1A, eIF2, eIF3), and the small ribosomal subunit. The mature mRNA transcript is recruited independently through the binding of the eIF4F complex to its 5′ end. The heterotrimeric eIF4F complex is formed by the association of the eIF4E, eIF4G, and eIF4A subunits, where eIF4E is responsible for the recognition and specific binding to the m^7^GTP cap found on the mRNA 5′ end. The 43S PIC is driven to the mRNA 5′ end through an interaction between the multisubunit eIF3 complex, and the eIF4G subunit of eIF4F. eIF4G also binds to both eIF4E and eIF4A, organizing the whole eIF4F complex, while eIF4A is an RNA helicase responsible for unwinding any secondary structures found in the mRNA’s 5′ untranslated region (5′ UTR). Simultaneously, the poly-A binding protein (PABP) binds to the mRNA’s 3′ poly-A tail, and through its interaction with eIF4G, promotes the mRNA circularization, leading to a closed-loop formation that enhances translation. The eIF4F helicase activity facilitates the motility of the whole PIC along the mRNA, a requirement for the scanning of the 5′ UTR, and the identification of the first AUG that defines the start of the open reading frame (i.e., protein-coding region). Following AUG initiation, the eIF4F complex is released, followed by the recruitment of the large ribosomal subunit, and subsequent formation of the first peptide bond of the nascent protein (see [33,34,35] for a comprehensive review about the translation process).

### eIF4E Family Members, Structure, and Function

The cap binding protein eIF4E is now recognized as representing a large eukaryotic protein family, generally defined by the presence of up to eight conserved tryptophan/aromatic residues. These were originally described based on the mammalian and yeast eIF4E homologs, with the mammalian sequence numbering used as reference here: W43, W46, W56, W73, W102, W112, W130, and W166. Four of these residues are essential for classical eIF4E functions, with W56, W102, and W166 being required for cap recognition, while W73 is involved in eIF4G binding [36]. By comparing eIF4Es from a number of organisms, it was possible to determine that these tryptophan residues are spaced within a conserved central region that is in agreement with the consensus H(X5)W(X2)W(X8–12)W-(X9)F(X5)FW(X20)F(X7)W(X10)W(X9–12)W(X3435)W(X32–34). This central region constitutes the typical eIF4E “core” found in all of this protein family members [37].

The eIF4Es fold into a “cupped-hand” tertiary structure, having eight antiparallel β-sheets and three α-helices, that is critical for its ability to bind to the m^7^GTP cap [38]. Cap binding is mediated by the two conserved tryptophans localized on eIF4E’s concave side, W56 and W102 (mammalian eIF4E1 numbering), required to “capture” the mRNA cap through a π–π stacking interaction. Binding interactions are also mediated through hydrogen bonds between E103 and the m^7^G cap, and by electrostatic interactions between the positively charged residues R112, R157, and R162, with the three phosphate groups of the m^7^GTP. A third tryptophan residue, W166, localized towards the C-terminus of eIF4E is also involved in a specific interaction with the 7-methyl moiety of the cap structure [39,40,41,42]. A network of hydrogen bonds also strengthen the eIF4E-cap interaction involving the residues W56, D90, F94, W102, E103, R112, N155, R157, K162, W166, and H200 [43]. Through its convex side, eIF4E may bind to several proteins with different roles, but its main partner in translation is eIF4G [44,45]. The eIF4E–eIF4G binding is mediated by the VEDFW residues from eIF4E and the canonical motif Y(X)_4_LΦ (where X represents any amino acid and Φ represents a hydrophobic residue) localized within the N-terminal third of eIF4G [46]. This motif is also found in proteins, like the well-known eIF4E-binding proteins (4E-BPs) [45], from several organisms that bind competitively to eIF4E, preventing it binding to eIF4G, either to promote translation or the translational arrest of specific mRNAs. A secondary non-canonical eIF4E binding motif within eIF4G, GLPHISDVVL in the human protein, also acts in stabilizing the eIF4E–eIF4G binding, by interacting with the hydrophobic residues F47, I63, L75, and I79 on the human eIF4E [47,48].

In higher eukaryotes, eIF4E is distributed mainly throughout the cytoplasm, although small amounts are present in the nucleus, where it is distributed in discrete spherical bodies. In the nucleus, eIF4E has been found to associate with the leucine rich pentatricopeptide repeat protein (LRPPRC), as part of a specific protein–RNA complex, known as ribonucleoparticle (RNP). The LRPPRC protein recognizes a ~50 nucleotide secondary structure element, called eIF4E sensitivity element (4E-SE), found along the 3′UTR of selected transcripts. This complex also associates with the RNA helicase UAP56 and with hnRNPA1 protein. Altogether the RNP acts in the export to the cytoplasm of over 700 selected transcripts, transiting through the nuclear pore complex via the CRM1 export receptor [49,50]. In the cytoplasm, eIF4E is also found in different types of cytoplasmic bodies, known as processing bodies (P-bodies) and stress granules (SG), that are respectively involved in mRNA decay or storage [51,52,53].

The major role of eIF4E is in translation. Despite the overall conservation in the translation process throughout the various eukaryotic lineages, several taxa have evolved unique mechanisms associated with mRNA recognition and ribosome recruitment during the initiation of translation of specific sets of mRNAs. These are reflected in a substantial variation in the number of eIF4E homologs among different organisms. It is thought that during evolution, a single ancient eIF4E gene underwent a series of gene duplications and modifications generating the families of related proteins seen in different eukaryotic organisms. Not all eIF4E homologs necessarily act as translation initiation factors [36]. A phylogenetic analysis made with eIF4E homologs from several organisms allowed their classification into three main groups: **eIF4E Class 1**, represented by the prototypical eIF4E and having the eight typical tryptophan residues, is able to bind to both the cap structure and eIF4G; **eIF4E Class 2**, represented by eIF4Es with substitutions in residues equivalent to W43 and W56 for another aromatic residue, which are involved in cap binding or 4E–BP interactions; and **eIF4E Class 3**, represented by proteins having the W56 residue replaced by a C or Y, with no function clearly described [37]. The major diversity of eukaryotic species, however, resides within protists, whose eIF4E homologs do not match this classification. The eIF4E from protists are instead distributed in three distinct clades, with functional data still limited to very few species [36].

Multiple eIF4E homologs and accessory proteins are, thus, commonly found in different organisms performing distinct roles in translation [44,45,54,55]. For instance, initial data indicated that mammalian eIF4E1 (Class 1) binds the cap, eIF4G homologs, and the 4E-BPs translation repressors, while eIF4E2 (Class 2) binds only the cap and 4E-BPs. In contrast, eIF4E3 (Class 3) binds the cap, eIF4G1 and eIF4G3 [53,56,57]. It has been thought for some time that eIF4E1 was the only eIF4E protein involved in translation initiation in mammals, while eIF4E2 (formerly 4EHP) and eIF4E3 would be involved in translation repression [58,59]. However, additional data indicated that mammalian eIF4E2 binds to eIF4G3 and translates a subset of mRNAs under hypoxic conditions [60], and that eIF4E3 is active in translation under MNK-kinase dependent inhibition, ablating eIF4E1 phosphorylation and promoting a unique translatome [61]. Hence, both eIF4E2 and eIF4E3 have roles in the translation of subsets of mRNAs under specific situations, by forming alternative eIF4F complexes [62]. Specific selection of mRNAs for different eIF4F complexes is also found in *Caenorhabditis elegans*, where five eIF4E homologs (from Classes 1 and 2) are differentially expressed and recognize specific subsets of mRNAs during the nematode development [63]. In plants, two eIF4F complexes, eIF4F and eIF(iso)4F (both classified as Class 1), may have different translation efficiencies and preferences for distinct mRNA subsets [64,65,66]. Bearing in mind the plethora of eIF4E functions that are associated with the control of translation of functionally related mRNAs, and considering that in trypanosomatids, subsets of mRNAs also seem to be grouped into post-transcriptional regulons with common regulatory mechanisms [67], it is a sound hypothesis that multiple eIF4E homologs may play a significant role in mRNA selection for translation as part of distinct eIF4F(-like) complexes (see Section 3 below).

## 3. eIF4E Homologs in Trypanosomatids and Protein Partners

Multiple homologs for the eIF4F subunits were identified in characterization studies involving mainly *Trypanosoma brucei* and *Leishmania* species. There are six eIF4E, five eIF4G, and two eIF4A homologs conserved in all trypanosomatid species investigated so far [68,69]. The eIF4E homologs vary significantly in size and properties, and will be discussed in detail below. The eIF4Gs also vary significantly in size and in the relative location of the conserved HEAT1/MIF4G domain, typical of all eIF4G homologs. Based on sequence comparisons with better known metazoan and yeast eIF4Gs, no clear motifs involved in the interactions with the eIF3 complex or with eIF4E and/or PABP homologs can be found [69], although they are able to interact with other putative eIF4F subunits, forming eIF4F(-like) complexes. For eIF4A, both homologs are important for cell survival, but only one is active during translation [70]. Additionally, there are two PABP homologs in *T. brucei* and three in *Leishmania* that may be involved in the differential selection of mRNAs [71,72]. In the initial report for the first trypanosomatid eIF4E, from *L. major*, the name LeishIF4E-1 was originally used [73]. However, we rather follow and use here the recommended nomenclature for trypanosomatid proteins [74], where the protein is named with a prefix representing the species abbreviation followed by the name of the protein in upper case (e.g., LmEIF4E1, TbEIF4E1). Alternative terminology found in the literature includes the protein name only and the species (e.g., *L. major* EIF4E1, *T. brucei* EIF4E1), or just the protein name, when referring to the archetype properties shared by the orthologs (e.g., EIF4E1, referring to both *L. major* and *T. brucei*).

The characterization of the first four eIF4E homologs in *T. brucei* classified them into two groups, based on structural and molecular properties [75]. With the identification of two more eIF4E homologs [68], however, a third group was proposed. **Group 1** then is formed by EIF4E1 and EIF4E2, relatively low abundant proteins with similar size and higher homology to the human eIF4E homolog, that cannot form eIF4F-like complexes nor are required for general translation. The eIF4E **Group 2** is formed by EIF4E3 and EIF4E4, more abundant proteins having long N-terminal extensions and lower overall homology within the eIF4E core, that however can form typical eIF4F complexes implicated in general translation and essential for cell survival. **Group 3** consists of EIF4E5 and EIF4E6, very small proteins with low eIF4E core homology, but nevertheless, able to bind to eIF4G homologs as part of novel eIF4F(-like) complexes unlikely to be involved in general translation [76]. As seems to be the rule for the more divergent eIF4Es from protists, the trypanosomatid eIF4E groups do not fit into the three previously defined eukaryotic eIF4E classes [37]. Nevertheless, further analysis comparing only eIF4E sequences from various protist groups confirm that the EIF4E1–EIF4E2 and EIF4E3–EIF4E4 pairs belong to phylogenetically independent eIF4E clades [36], thus confirming their separation, based on their biological properties, into two independent groups [75]. The detailed description of these three trypanosomatid eIF4E groups will be considered now, taking into account the following parameters commonly used to study eIF4E homologs: in silico structural properties, analysis of cap-binding affinities, protein expression levels, binding partners and formation of eIF4F(-like) complexes, and potential involvement during translation [77,78].

### 3.1. Trypanosomatid eIF4E Group 1: EIF4E1 and EIF4E2

The first reported eIF4E homolog from trypanosomatids, LmEIF4E1 (GeneDB ID#: LmjF.27.1620), was characterized from *L. major* and described as a 214 aa protein (23.84 kDa) with 42% similarity to mammalian eIF4E. Its predicted 3D structure, modeled using alternatively the GenTHREADER and Modeller programs or the SwissPDBViewer, indicated that it should fold into a structure overall compatible to the one described for the mammalian eIF4E, despite the low conservation in sequence between the two proteins. Exceptions are the two short insertions inside the eIF4E core region, that could not be modeled, and the lack of the C-terminus found in both mouse and yeast homologs [69,73]. These initial reports emphasized the main features of LmEIF4E1, where the conservation of seven out of the eight characteristic tryptophan residues typical of eIF4E family members [40,57] was observed, including those involved in cap binding: W37, W93, and W176 (equivalents to W56, W102, and W166 in mammals). The single exception was the F54 residue replacing the equivalent W73 position of mammalian eIF4E, involved in the interaction with eIF4G. Counterparts to several other residues involved in known eIF4E interactions (such as E103, R112, R157, and K162 in the mammalian protein) were also found in LmEIF4E1 (E84, K93, R167, and K172) [69,73]. TbEIF4E1, the *T. brucei* EIF4E1 ortholog (GeneDB ID#: Tb927.11.2260) was later characterized as a 233 amino acid polypeptide (26.02 kDa) with characteristics conserved with the *Leishmania* protein, and also having seven out of the eight conserved tryptophan residues, with a phenylalanine replacing W73 [75].

**Figure 1:**
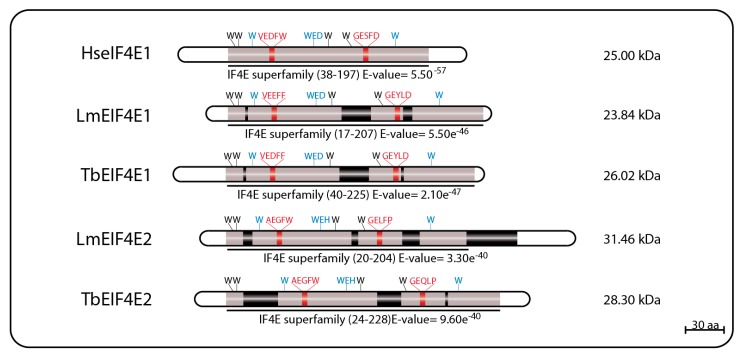
Schematic representation of EIF4E1 and EIF4E2 homologs from *T. brucei* and *L. major*. The eIF4E core regions are represented in grey; black boxes represent insertions within these core regions; tryptophan residues involved in cap binding are highlighted in blue; residues involved in eIF4G-binding are highlighted in red. The E-value numbers and the domain boundaries were generated through sequence searches using the on line tool Pfam Database [80,81].

LmEIF4E2 (GeneDB ID#: LmjF19.1500/LmfF19.1480) is a 281 aa protein (31.46 kDa) with 41% similarity to the human homolog and whose gene is found in two copies within the *L. major* genome. This protein has all conserved tryptophan residues typical of eIF4E homologs, including those equivalent to W56, W102, and W166 in the human protein involved in cap binding (W45, W91, and W173 in LmEIF4E2), as well as positively charged residues required to bind to the phosphate groups of the m^7^GTP (K101, R152, and K169). Noteworthy is the replacement of a highly conserved aspartate in eIF4E sequences, D104 in the human protein, for a histidine (W93). LmEIF4E2 is also characterized by the presence of short insertions localized within the eIF4E core region, next to amino acids relevant to cap binding, and a much larger insertion closer to its C terminus, but its 3D structure was also compatible with mammalian eIF4E [69,79]. Its *T. brucei* ortholog, TbEIF4E2 (GeneDB ID#:Tb927.10.16070), is a 251 aa protein (28.3 kDa) with features similar to those found in LmEIF4E2, but with changes the insertions found within the LmEIF4E2 core and lacking C-terminal extension entirely [75]. A schematic representation of the two sets of EIF4E1 and EIF4E2 homologs described here can be seen in Figure 1, with relevant sequence motifs in common highlighted.

#### 3.1.1. EIF4E1 and EIF4E2 Subcellular Distribution and Abundance

Early cell fractionation assays, analyzed by Western blotting, determined that both LmEIF4E1 and LmEIF4E2 were found mainly in cytoplasmic fractions [73,79]. Later, indirect immunofluorescence using specific antibodies for the native proteins, and direct visualization of ectopically expressed fluorescent TbEIF4E1 and TbEIF4E2, fused to EYFP, indicated that they can be found in both the cytoplasm and nucleus, at least in *T. brucei* procyclic cells [75,82]. Under standard growth conditions, TbEIF4E1 was also seen to partially localize to cytoplasmic granules and under nutritional deprivation and sinefungin treatment; a large fraction co-localized with the stress granule marker DHH1 within granules localized in the nuclear periphery and within sinefungin-induced p-bodies, exhibiting a behavior similar to that found for PABP2. In the case of TbEIF4E2, only a minor presence was detected in starvation stress granules [72]. More recent data, derived from the whole proteome tagging project for the fluorescence localization of *T. brucei* proteins, indicates that TbEIF4E1 is found mainly in the cytoplasm, within various speckles, with no apparent presence in the nucleus (data available at Tryptag [83]). 

Expression analysis by Western blotting in promastigote cell extracts have shown that LmEIF4E1 is not an abundant protein, found in roughly 2 to 4 × 10^3^ molecules/cell in *Leishmania* [69] and 3 to 8 × 10^3^ molecules/cell in *T. brucei* procyclic form and 1.5–5 × 10^3^ molecules/cell for bloodstream form [75]. In *L. amazonensis*, Western blotting assays using cell extracts from promastigotes and amastigote-like forms indicated that LaEIF4E1 is expressed more in amastigotes [79]. This higher expression was postulated to be due to an element found within the 3′UTR of the LmEIF4E1 mRNA that is similar to the regulatory element found in the *Leishmania* amastin messenger, and is involved in amastigote-specific expression [79,84]. LmEIF4E2 is also not abundant, detected at about 10^3^ molecules per cell in *Leishmania* [69] and between 1 to 5 × 10^3^ molecules/cell in both forms of *T. brucei* [75]. Microarray analysis has detected LmEIF4E2 within a subset of genes expressed preferentially in metacyclic form of *L. major* [85], but to date, this has not been confirmed by protein expression analysis.

#### 3.1.2. EIF4E1 and EIF4E2 Cap Binding Affinities

Cap binding affinity assays have shown that recombinant LmEIF4E1 is able to bind to ^7^mGTP Sepharose 4B beads, and can be specifically eluted with soluble m^7^GTP. Additionally, time-synchronized fluorescence titration assays have shown that LmEIF4E1 is able to bind to both cap4 and m^7^GTP, in contrast to mouse eIF4E1, which has lower affinity to cap4 than to m^7^GTP [69,73,79]. Its *T. brucei* ortholog, TbEIF4E1, was only tested with the m^7^GTP resin, but its binding to the beads was very similar to what was seen with LmEIF4E1 [75]. In contrast, LmEIF4E2 failed to bind efficiently to the m^7^GTP beads [69], even though it has all residues required for such interaction, and the fluorescence titration assay revealed that it has a much greater affinity to cap4 when compared with m^7^GTP and other cap analogs [79]. TbEIF4E2 differs markedly from its *Leishmania* ortholog in that it was able to bind efficiently to the m^7^GTP beads, a difference that might be due to structural differences associated with the unique LmEIF4E2 insertion seen next to its C-terminus [75].

#### 3.1.3. EIF4E1 and EIF4E2 Partners and Possible Roles in Translation

**Figure 2:**
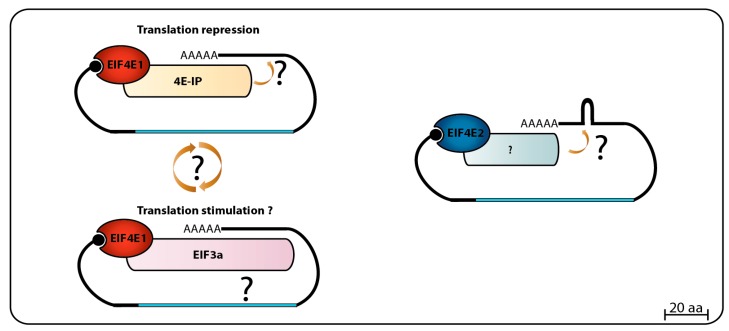
Summary of the complexes formed EIF4E1 and EIF4E2. Available data indicates that EIF4E1 might be involved in translation repression when bound to 4E-IP, and translation stimulation when bound to EIF3 complex through EIF3a subunit interaction. EIF4E2 interaction with its specific partner and their biological function still needs to be addressed.

When *Leishmania* whole-cell lysates were loaded onto m^7^GTP-Sepharose columns, and specifically bound proteins eluted with soluble cap analogue, LmEIF4E1, LmEIF4E4, LmEIF4G3, and LmEIF4AI were found in the eluted fraction. This was interpreted as both eIF4E homologs were able to interact with LmEIF4G3 and LmEIF4AI, and indeed, GST pull-down assays seemed to confirm an interaction between LmEIF4G3. Also, LmEIF4E4, LmEIF4A1 and a small amount of LmEIF4E1 came down with the eIF4G homolog when a FLAG-tagged LmEIF4G3 was specifically precipitated. Polysomal fractionation analysis using sucrose gradients identified both LmEIF4E1 and LmEIF4E4 in heavier fractions co-migrating with LmEIF4G3 in lysates made from regular promastigotes grown at 26 °C, but the polysomal presence of LmEIF4E4 decreased in lysates made with parasites exposed to elevated temperatures, therefore mimicking vertebrate host conditions, while LmEIF4E1 could still be detected in the same fractions. These results led to the conclusion that the LmEIF4E1–LmEIF4G3 complex could be active in translation in both parasite life stages [86]. However, follow up mass spectrometry and yeast two-hybrid assays refuted the interaction between LmEIF4E1 and LmEIF4G3, and between LmEIF4E1 and any of the remaining *Leishmania* eIF4G homologues [87]. Subsequent in vitro pull-down assays also failed to confirm the interaction between recombinant LmEIF4E4 and LmEIF4E1, and in *T. brucei*, in vitro and in vivo assays confirmed that LmEIF4E4 is the true and only LmEIF4G3 eIF4E partner [75,88]. The *Leishmania* mass spectrometry data, however, did identify a novel EIF4E1 partner, called 4E-interacting protein (4E-IP). This is a mostly unstructured protein lacking homologs outside the trypanosomatids, but that seems to interact with EIF4E1 through a canonical eIF4E binding motif, **Y**TREE**LL** [87]. 4E-IP is constitutively expressed in *Leishmania* [89], but its interaction with EIF4E1 seems to be restricted to the promastigote stage of the parasite life-cycle, since it was not detected in *L. amazonensis* amastigote-like forms [87]. More recently, it was reported that LmEIF4E1 interacts directly with the *Leishmania* translation initiation complex eIF3, through the C-terminus region of the EIF3a subunit, indicating a role in translation independent of any eIF4G function [90]. In *T. brucei* TbEIF4E1, and also TbEIF4E2, knockdown assays in procyclic cells did not show any particular effect on cell growth/viability or any effect in translation, but TbEIF4E1 knockdown in bloodstream cells did lead to a decrease in cell growth, but without compromising cell viability, while TbEIF4E2 knockdown did not cause any apparent growth effect [75]. High throughput RNAi assays have shown only abnormal cell growth in late bloodstream form for both proteins [91]. In contrast, double knockdown of TbEIF4E1 and TbEIF4E2 leads to a rapid cell death in procyclic cells, with no significant effect on translation, while double knockdown of TbEIF4E1 and TbEIF4E4 leads to cell growth arrest with a major reduction in translation, but no immediate cell death [75]. A major breakthrough in sorting the TbEIF4E1 function came from a screening using a tethering assay aimed at identifying potential activators and repressors of mRNA translation. Both TbEIF4E1 and the Tb4E-IP ortholog were seen to efficiently suppress the expression of a reporter mRNA when tethered to its 3′UTR, indicating a role for both proteins in translation repression [92,93]. Figure 2 summarizes the data available so far regarding EIF4E1 function in trypanosomatids, with potential roles in both translation stimulation and repression. Much less is known about EIF4E2, however, our unpublished work indicates a strong physical association of TbEIF4E2 with the histone mRNA stem-loop binding protein.

### 3.2. The Trypanosomatid eIF4E Group 2: EIF4E3 and EIF4E4

LmEIF4E3 (GeneDB ID#: LmjF.28.2500) is a 349 aa protein (38.0 kDa) with 43% similarity to human homologs, having only four of the conserved tryptophan residues typical of eIF4E sequences. These include the residues equivalent to W102 and W166, involved in cap binding (W216 and W286 in LmEIF4E3), and the equivalent to the mammalian W73 residue involved in binding to eIF4G (W187 in LmEIF4E3). The third tryptophan involved in cap recognition, W56, is replaced by a phenylalanine (F172), but several positively charged amino acids are also found near to positions equivalent to R112, R157, and K162 in human eIF4E. As for EIF4E2 sequences, the near universal aspartate at position 104 in the mammalian eIF4E, next to or within the cap binding pocket, is replaced by a histidine in LmEIF4E3 (H218) and in other *Leishmania* EIF4E3 orthologs. TbEIF4E3 (GeneDB ID#: Tb927.11.11770) is a 442 aa protein (47.96 kDa) also having four conserved tryptophan residues at equivalent positions as well as similar positively charged amino acids and the D104 replacement. The feature common to the various EIF4E3 sequences, which distinguish them from other eIF4Es, is their long N-terminal extension, also found in EIF4E4 homologs (see below) [69,75,94].

**Figure 3:**
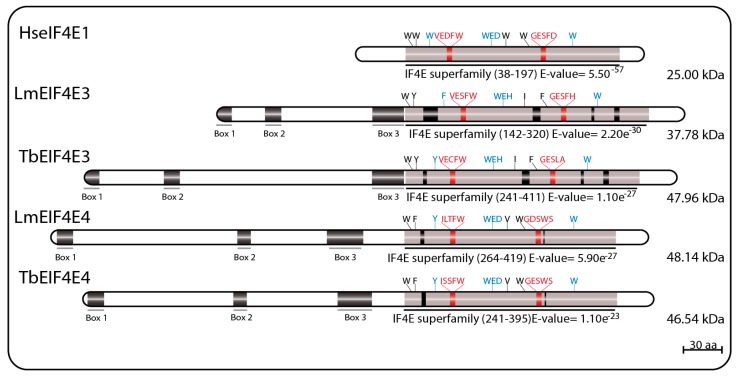
Schematic representation of EIF4E3 and EIF4E4 homologs from *T. brucei* and *L. major*. The eIF4E core regions are represented in grey; insertions within these core regions are represented by black boxes; tryptophan residues involved in cap binding are highlighted in blue; and residues involved in eIF4G-binding are highlighted in red. The Boxes 1, 2 and 3 represent three conserved regions likely involved in binding to PABP homologs and localized within the N-terminal extensions of EIF4E3 and EIF4E4. The E-value numbers and the domain boundaries were generated through sequence searches using the on line tool Pfam Database [80,81].

LmEIF4E4 (GeneDB ID#: LmjF.30.0450) is a 447 aa protein (48.0 kDa) having 45% similarity to the human homolog. Five of the conserved tryptophan residues typical of eIF4Es are present including those equivalent to both W102 and W166 (W330 and W391 in LmEIF4E4), involved in cap recognition, and the W73 residue required for the eIF4G interaction (W302). As for LmEIF4E3, the third cap-interacting tryptophan in humans, W56, is replaced by another aromatic residue in LmEIF4E4 (a tyrosine, Y287), but positively charged amino acids required to stabilized the cap interaction are generally also present [69]. TbEIF4E4 (GeneDB ID#: Tb927.6.1870) is a 427 aa protein (46.54 kDa) also having the same five conserved tryptophan residues and related residues in the positions described above [75]. Additionally, similar to LmEIF4E3, long N-terminal extensions are also present in all trypanosomatid EIF4E4 orthologs, although little homology in sequence is observed between the EIF4E3 and EIF4E4 extensions [69,75,94]. Major sequence features for both sets of EIF4E3 and EIF4E4 homologs are represented in Figure 3.

#### 3.2.1. EIF4E3 and EIF4E4 Subcellular Distributions and Abundance

Cell fractionation assays were also used for the preliminary definition of the LmEIF4E3 and LmEIF4E4 subcellular localization, and both were found to be present exclusively in the cytoplasm in promastigotes [79]. This was confirmed for the *T. brucei* orthologs using immunofluorescence with polyclonal serum directed against the two native proteins, as well as direct fluorescence assays using EYFP tagging in procyclic cells [75,82]. The same localization was seen for their respective eIF4G partners, TbEIF4G4 and TbEIF4G3 [88] (see below). Results from the whole proteome fluorescence tagging project confirmed the conspicuous cytoplasmic localization of TbEIF4E3 with no granular formation under standard growth conditions [83]. Further immunolocalization experiments in *Leishmania*, however, revealed that under starvation conditions, LmEIF4E3 migrated to cytoplasmic granules that apparently lacked its partner LmEIF4G4. Based on these and other results, a hypothesis has been proposed that LmEIF4E3 is inactive under optimal growth conditions when bound to LmEIF4G4, but upon phosphorylation, under nutrient deprivation, dissociates from LmEIF4G4 and enters stress granules where inactive mRNAs are stored [95]. This hypothesis, however, is not supported by the data from *T. brucei*, where TbEIF4E3 exhibited only minor localization to stress granules, a pattern also seen for TbEIF4E4, TbEIF4G3, and TbPABP1 [72].

The quantification of the EIF4E3 expression has shown that it is the most abundant of the eIF4E homologs in trypanosomatids, with its abundance estimated to range within 2 × 10^4^ and 10^5^ molecules/cell for *Leishmania* promastigotes, as well as both procyclic and bloodstream life forms of *T. brucei*. EIF4E4, with its abundance quantified only in *T. brucei*, was found to be the second most abundant eIF4E homolog, ranging from 2 to 4 × 10^4^ and 1 to 2 × 10^4^ molecules/cell in procyclic and bloodstream forms, respectively [69,75].

#### 3.2.2. EIF4E3 and EIF4E4 Cap Binding Affinities

Cap binding assays using ^7^methyl-GTP Sepharose 4B beads determined that neither LmEIF4E3 nor TbEIF4E3 were able to bind to the monomethylated cap structure found in m^7^GTP-Sepharose beads [69,75]. Time-synchronized fluorescent titration assays, however, established that LmEIF4E3 is able to bind to the m^7^GTP, and that its affinity is significantly higher than to the cap4 analogues [79]. In contrast, LmEIF4E4 was found to have the highest affinity to both m^7^GTP and cap4 analogues among the various trypanosomatid eIF4Es [68,79,96]. Indeed, m^7^GTP-Sepharose beads were used to efficiently purify native LmEIF4E4 from whole soluble *Leishmania* cytoplasmic fractions [86], the recombinant protein from bacterial extracts [79], and the ^35^S-labelled LmEIF4E4 after in vitro translation in the rabbit reticulocyte system [75]. Cap binding analysis by TbEIF4E4 also confirmed a similar affinity to m^7^GTP-Sepharose beads as found in LmEIF4E4 [75]. Altogether, the data collected indicate that EIF4E4 has a cap binding affinity substantially higher than EIF4E3.

#### 3.2.3. EIF4E3 and EIF4E4 Partners and Binding Interactions

The first trypanosomatid eIF4F-like complex was defined after the purification of both LmEIF4E4 and LmEIF4G3 from whole *Leishmania* cell lysates using m^7^GTP-Sepharose beads, followed by the confirmation that LmEIF4G3 is a true LmEIF4E4 partner through co-precipitation assays [86]. Mass spectrometry analysis of tagged LmEIF4E4 purified from lysates of *L. major* promastigotes confirmed that the complex LmEIF4E4–LmEIF4G3–LmEIF4AI occurs in vivo, and co-precipitates with LmPABP1 and LmPABP2, plus several eIFs and ribosomal proteins, reinforcing the model that LmEIF4E4 is part of a functional translation initiation complex acting in cooperation with LmEIF4G3, LmEIF4AI, and PABP homologs [87]. The interaction between EIF4E4–EIF4G3 homologs was also confirmed in *T. brucei* through pull-downs and immunoprecipitation assays, where tagged TbEIF4E4 brought down TbEIF4G3 and TbEIF4AI. The same report also defined a second trypanosomatid eIF4F complex through the identification of TbEIF4G4 as the TbEIF4E3 functional partner, both proteins co-precipitating with TbEIF4AI [75].

The direct binding between LmEIF4E4 and LmEIF4G3 was initially confirmed using the yeast two-hybrid system and found to require the motif **Y**PG**F**S**L**D (critical residues for eIF4E binding are highlighted), localized within the short LmEIF4G3 N-terminus [86]. Independent pull-down assays found that although the replacement of the FSL residues (F23/S24/L25) for three alanines caused ablation of the LmEIF4E4–LmEIF4G3 interaction, additional substitutions nearer to the LmEIF4G3 N-terminus and targeting two neighboring isoleucine and arginine residues (I8/R9) also prevented this interaction [88]. Within EIF4E4, additional yeast two-hybrid experiments have identified the LmEIF4E4 W302 (originally reported as W163 due to a genome annotation error) as critical for LmEIF4G3 binding [87]. The direct LmEIF4E3–LmEIF4G4 interaction was also confirmed through yeast two-hybrid assays and mass spectrometry analysis of proteins co-precipitated with tagged LaEIF4E3 and LaEIF4G4 in *L. amazonensis*, the two proteins likely forming a complex with LmEIF4A1 and LmPABP2. The EIF4E3–EIF4G4 interaction was seen to require the W187 from LmEIF4E3, and the conserved leucine L26 in LmEIF4G4 [95]. Further pull-down assays revealed that the LmEIF4E3 binding site in LmEIF4G4 indeed involves L26 plus the neighboring I25. Both residues align with I8/R9 from LmEIF4G3, and may form part of a divergent eIF4E binding motif, related to but distinct from the proposed consensus described within eIF4G homologs from higher eukaryotes (YXXXXLΦ—X meaning any amino acid residue, while Φ indicates a long-chain hydrophobic residue such as L, M, or F [56]). The F23/S24/L25 residues from LmEIF4G3 also implicated in its binding to LmEIF4E4 align with an equivalent motif in LmEIF4G4 as well (F23/S24/L25), however, their replacement for three consecutive alanines does not impact on the EIF4E3–EIF4G4 interaction [88].

**Figure 4:**
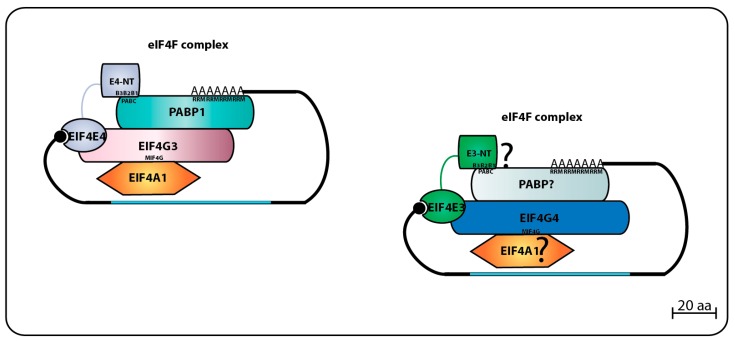
Summary of eIF4F complexes formed by EIF4E4 or EIF4E3 and their respective partners. EIF4E4 forms the main eIF4F complex in trypanosomatids involved in translation by interacting with EIF4G3 (which also binds to EIF4A1), and to PABP1 through three small regions (Boxes B1, B2 and B3) at its N-terminal extension which bind to the PABPC domain of PABP1. Similar composition might perhaps be found on the secondary eIF4F complex formed by EIF4E3 with EIF4G4, EIF4A1 and a PABP homologue.

Through the study of their eIF4G partners, vital aspects of the trypanosomatid eIF4E functions have been clarified. Both LmEIF4G3 and LmEIF4G4 were found to be capable of binding to EIF4AI, the only eIF4A homolog implicated in translation initiation in trypanosomatids [70]. This interaction is mediated by the eIF4G HEAT1-MIF4G domain, and requires the conserved L67/N68/K69 motif (LmEIF4G3 numbering). The HEAT1-MIF4G domain from LmEIF4G3 binds to LmEIF4AI on its own, but for LmEIF4G4, this interaction requires the full-length protein [94]. LmEIF4G3 can also interact with LmPABP1 in vitro, and specifically co-precipitates with native LmPABP1, but not with LmPABP2 nor LmPABP3 [71,88]. The direct LmEIF4G3–LmPABP1 interaction, however, could not be confirmed by yeast two-hybrid assays [87]. Surprisingly, subsequent yeast two-hybrid experiments reported a new specific interaction between LmEIF4E4 and LmPABP1, involving the LmEIF4E4 N-terminal extension [87]. This unique EIF4E4–PABP1 interaction in *L. infantum* was later defined to be dependent on three conserved motifs that fit the consensus L/MN/DXXAXXY/FXP localized at the N-terminus of EIF4E4, and it is essential for cell viability [94] (the three conserved consensus sequences or boxes are represented in Figure 4 below labeled as B1, B2, and B3). The consensus that emerges then is that the EIF4E4–EIF4G3 based complex is tightly associated with PABP1, and these proteins have common roles in translation. At this stage, however, it is not clear if a similar association exists between the second eIF4F-like complex, EIF4E3–EIF4G4, and PABP2. Figure 4 summarizes the data available regarding the two eIF4F-like complexes based on EIF4E3 and EIF4E4 and their binding partners.

#### 3.2.4. EIF4E3 and EIF4E4 Roles in Translation

So far, among all trypanosomatid eIF4E homologs, only EIF4E3 and EIF4E4 have been implicated as having direct roles in translation. Early sucrose gradient analysis had already shown that both LmEIF4E3 and LmEIF4E4 were present in polysomes [79,86], a result corroborated by more recent data that has found LmEIF4G3, the EIF4E4 partner, interacting directly with the multisubunit eIF3 complex, therefore highlighting the role of the LmEIF4E4–LmEIF4G3–LmEIF4AI–LmPABP1 assembly as a participant in translation initiation [90,97,98]. For EIF4E3, however, conflicting results were derived from *L. amazonensis*, where it was not found associated with the polysomal fractions, but instead, was found mainly in the top fractions of the gradient [95]. In contrast, in *T. brucei*, both TbEIF4E3 and TbEIF4E4 and their corresponding partners, TbEIF4G4 and TbEIF4G3, as well as TbEIF4AI, were detected in free and membrane-bound polysomes, with TbEIF4E4 the most abundant in polysomes [99]. In agreement, the tethering screen performed in *T. brucei* to search for activators and repressors of mRNA translation defined both TbEIF4E3 and TbEIF4E4 as well as their partners, TbEIF4G4 and TbEIF4G3, and the two PABP homologs (TbPABP1 and TbPABP2) as activators capable of enhancing the translation of a reporter mRNA when tethered to its 3′UTR [92,93].

The implications for a role in translation for both EIF4E3 and EIF4E4 are reinforced by the results derived from RNAi in experiments in *T. brucei*. Induced, RNAi based, TbEIF4E3 knockdown leads to cell death in both procyclic and bloodstream forms of *T. brucei*, with an apparent direct effect in translation, at least in procyclic cells [75]. A similar lethal effect was also found for TbEIF4G4 after depletion in procyclic cells, but in contrast to its partner, this was not associated with significant decreases in protein synthesis [88]. These results were independently confirmed by high throughput RNAi experiments, where loss of either TbEIF4E3 or TbEIF4G4 led to significant loss of fitness in procyclic, bloodstream, and differentiation stages of *T. brucei* [91]. In contrast, TbEIF4E4 knockdown prevents viability only in bloodstream forms, although a nearly complete arrest in translation and growth, without cell death, is observed when both TbEIF4E4 and TbEIF4E1 are simultaneously depleted in procyclics [75]. The data for TbEIF4G3 is more robust, since its depletion in procyclic forms leads to a very rapid arrest in protein synthesis, without apparent morphological implications and preceding cell death [88]. Interestingly, in the independent high throughput RNAi experiments, depletion of TbEIF4E4 leads to reduced growth in late bloodstream stage and mainly affected differentiating cells, while TbEIF4G3 depletion produced no effect [91]. Regardless of the lack of RNAi effect in *T. brucei* procyclic cells, the *EIF4E4* gene cannot be deleted in *L. infantum*, and complementation assays have shown that the LiEIF4E4–LiPABP1 binding is required for LiEIF4E4 function and cell viability. Cells having a mutant LiEIF4E4 unable to interact to LiEIF4G3 as the sole LiEIF4E4 source were viable, but could not differentiate to amastigotes [94]. Another implication for the importance of TbEIF4E4 function during differentiation was the RNA-dependent association of TbEIF4E4 with PTP-tagged ALBA2 and ALBA3 in *T. brucei* [100]. ALBA proteins are involved in several cellular processes that include genome packaging and organization as well as transcriptional and translational regulation [101]. Downregulation of ALBA3 and ALBA4 is apparently involved in trypanosome development and differentiation from mesocyclic trypomastigotes to the epimastigote stage in the anterior midgut of tsetse fly [102]. Remarkably, double RNAi of the ALBA3/ALBA4 proteins produced a cell growth arrest similar to the one observed for the TbEIF4E1/TbEIF4E4 double RNAi, also leading to a morphologically similar, elongated cells [75,102].

#### 3.2.5. EIF4E3 and EIF4E4 Phosphorylation

In *L. amazonensis*, under conditions of nutrient depletion emulating the environment inside the insect midgut, EIF4E3 has been shown to undergo hyperphosphorylation, with a likely impact on the LaEIF4E3–LaEIF4G4 interaction. In vitro differentiation assays have also shown that in axenic amastigotes, LaEIF4E3 undergoes hyperphosphorylation, and the LaEIF4G4 abundance was significantly reduced [95]. Multiple phosphorylation events targeting EIF4E3 and EIF4E4 were independently detected in both *Leishmania* and *Trypanosoma* species, and found to be mainly directed to their N-terminal extensions [94,103,104,105]. *L. amazonensis* expression analyses have shown that although LaEIF4E3 and LaEIF4E4 are constantly expressed during different growth phases, they show opposite phosphorylation patterns when log and stationary phase of growth cultures are compared. LaEIF4E3 seems to be preferentially phosphorylated early after passaging and during late, stationary phase growth. In contrast, multiple phosphorylated isoforms of LaEIF4E4 predominate during exponential growth, coinciding with active protein synthesis. This pattern of phosphorylation is conserved in *T. brucei* for TbEIF4E4, but not for TbEIF4E3 [103]. Similar results for LiEIF4E4 were also seen in *L. infantum* where phosphorylated LiEIF4E4 was present during active growth of both promastigotes and differentiated amastigotes, while the dephosphorylated LiEIF4E4 appeared in late stationary phase culture in both life stages. Through mutagenesis analysis, the LiEIF4E4 phosphorylation was studied further and found to be directed to multiple serine or threonine residues, followed by a proline (SP or TP motifs), a pattern of phosphorylation associated with MAP-like or CDK-like kinases. Contrary to what is seen in mammals, where the eIF4E kinase, Mnk, binds to eIF4G in order to phosphorylate eIF4E, the phosphorylation of LiEIF4E4 is not dependent on its interaction with LiEIF4G3. An LiEIF4E4 mutant missing most of the phosphorylation sites, however, can still be functional and replace the wild type protein in complementation assays [94]. More recently, in *T. brucei*, TbEIF4E4 and its TbPABP1 partner have been found to be phosphorylated by CRK1, a CDK kinase involved in controlling the G1/S transition [106].

### 3.3. The Trypanosomatid eIF4E Group 3: EIF4E5 and EIF4E6

EIF4E5 and EIF4E6 are the least studied and the more recently described of the trypanosomatid eIF4Es, having very limited sequence homology to other eIF4E homologs. In *L. major*, LmEIF4E5 (GeneDB ID#: LmjF.36.0590) is a small, 219 aa (23.9 kDa) protein having only four of the typically conserved tryptophan residues, including two equivalent to the mammalian eIF4E W56 and W166 involved in cap binding and recognition (W33 and W156 in LmEIF4E5, respectively) and the equivalent to the W73 implicated in eIF4G binding (W58). The third cap binding residue, W102 in mammalian eIF4E, is replaced by a conserved aromatic substitution (Y88). The *T. brucei* TbEIF4E5 ortholog (GeneDB ID#: Tb927.10.5020) is a 195 aa protein (21.9 kDa) also having the same four conserved tryptophan residues, and the aromatic substitution for the mammalian W102. Both proteins also have several positively charged residues at the positions required for the stabilization of the cap interaction [68].

**Figure 5:**
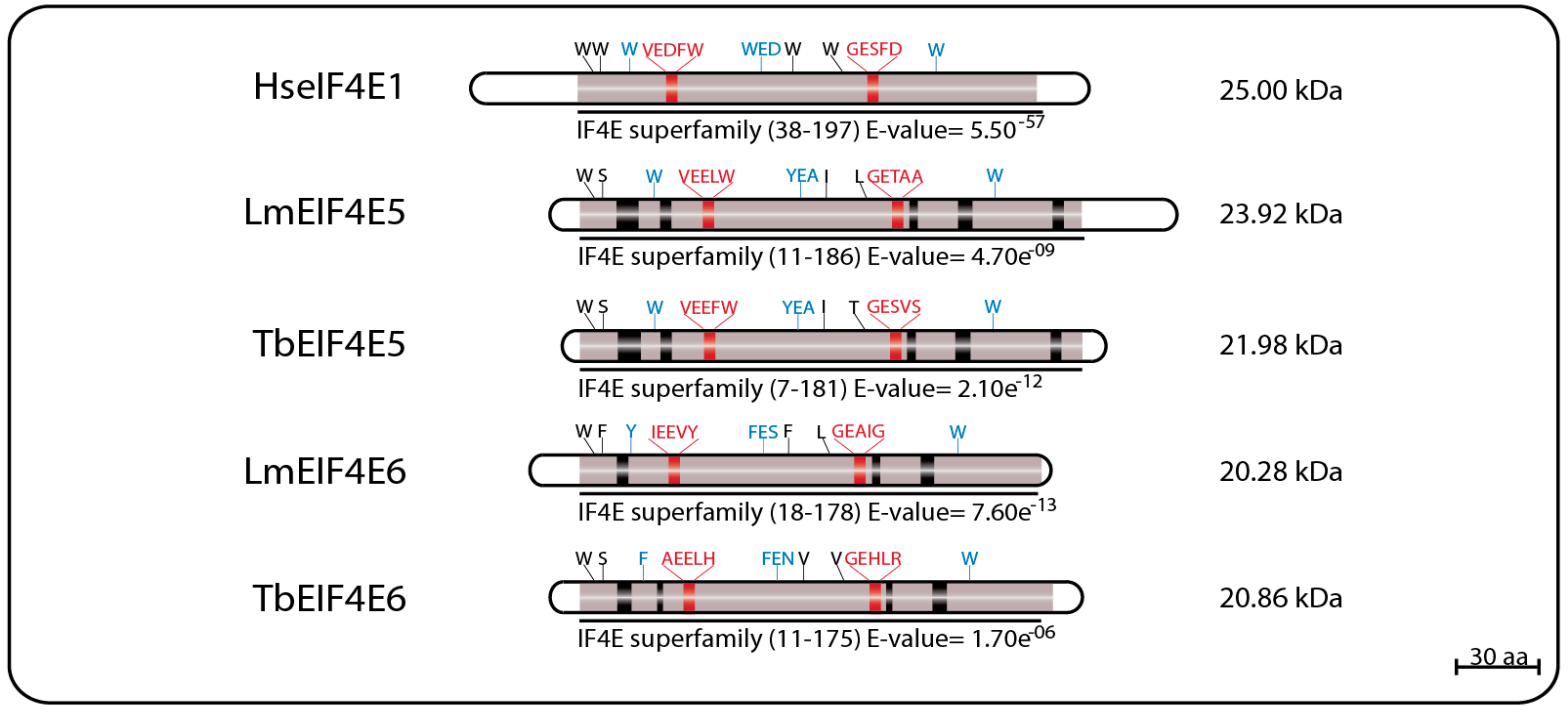
Schematic model of EIF4E5 and EIF4E6 homologues from *T. brucei* and *L. major*. eIF4E core region is represented in grey rectangle; Insertions inside the core region of EIF4E5 and EIF4E6 are represented in black boxes; Tryptophan residues involved in cap binding are highlighted in blue; Residues involved (or equivalent) in eIF4G-binding are highlighted in red. The E-value numbers and the domain boundaries were generated through sequence searches using the on line tool Pfam Database [80,81].

The EIF4E6 proteins are the smallest of the eIF4E homologs, with the 182 aa LmEIF4E6 (20.28 kDa—GeneDB ID#: LmjF.26.0240) having only two conserved tryptophan residues, including the one equivalent to W166 (W150 in LmEIF4E6), as well as conserved aromatic substitutions replacing the W56 and W102 cap-interacting residues (Y36 and F82), and the W73 involved in the eIF4G interaction (Y53). Its *T. brucei* ortholog, TbEIF4E6 (GeneDB ID#: Tb927.7.1670), is a 186 aa (20.86 kDa) protein having the same conserved tryptophan residues seen for its *L. major* counterpart, as well as aromatic substitutions replacing the W56 and W102 mammalian residues (F29 and F80). Noteworthy, however, is the lack of an aromatic residue at the W73 position, replaced by a histidine (H51) in TbEIF4E6. As for EIF4E5, both *L. major* and *T. brucei* EIF4E6 orthologs also have positively charged arginine and lysine residues in the positions required to stabilize the cap interaction [96]. Sequence features for both sets of EIF4E5 and EIF4E6 homologs are represented in Figure 5.

#### 3.3.1. EIF4E5 and EIF4E6 Subcellular Distributions, Abundance, and Cap Binding Affinities

With the lack of efficient antibodies to evaluate its expression, the cellular distribution of TbEIF4E5 was determined through immunofluorescence against using a PTP-tagged protein. Localization was found to be diffuse, throughout the cytoplasm, with numerous foci of greater intensity [68], similar to what was found for TbEIF4E3 and TbEIF4E4 [75,82]. Using a similar approach, TbEIF4E6 was also found as a cytoplasmic protein absent from the nucleus [96]. The results for both proteins are in agreement with the data from the whole proteome fluorescence tagging project [83]. 

No precise quantitative data for the abundance of EIF4E5 or EIF4E6 is available to date, but preliminary analysis revealed that both are the least expressed of the eIF4E homologues in the insect forms from both *L. major* and *T. brucei* (our group unpublished data). According to a high throughput proteomic expression analysis, LmEIF4E5 is constitutively expressed in *L. major* promastigotes and in lesion derived amastigote [89]. Despite their sizes, both EIF4E5 and EIF4E6 have structural features typical of cap-interacting proteins. To evaluate their cap binding affinities, they were also tested using fluorescence-titration curves with four different cap analogs. Recombinant TbEIF4E5 was found to bind to both m^7^GTP and cap4 with equivalent affinities to both analogs [68], falling within the range of other *L. major* eIF4Es [79]. TbEIF4E6 was also tested using the same assay, and it also binds similarly to both m^7^GTP and cap4, although with overall affinities lower than TbEIF4E5 [96].

#### 3.3.2. EIF4E5 Binding Partners

The search for putative TbEIF4E5 and TbEIF4E6 partners started with a yeast two-hybrid experiment designed to evaluate possible interactions with any of the five known trypanosomatid eIF4Gs from *T. brucei*. For TbEIF4E5, these assays revealed that it interacted specifically with two eIF4G homologs, TbEIF4G1 and TbEIF4G2, but with none of the other eIF4Gs tested. The binding was specific for TbEIF4E5, since neither of the two eIF4G homologs was able to interact with the remaining trypanosomatid eIF4Es. TbEIF4E5 binding to TbEIF4G2 seemed to be stronger than to TbEIF4G1, but the assay was not appropriate to evaluate quantitative differences. Mass spectrometry analysis with purified tagged TbEIF4E5 confirmed the TbEIF4E5–TbEIF4G1 and TbEIF4E5–TbEIF4G2 interactions in vivo. Tagging and purification of both eIF4Gs revealed the presence of TbEIF4E5 in two independent sub-complexes, each with a different eIF4G homolog. The first complex is formed by TbEIF4E5, TbEIF4G1, a homolog for the protein known as 14-3-3 (Tb927.11.68710), and two hypothetical proteins Tb927.11.6720 (117.5 kDa) and Tb927.11.350 (47.5 kDa). The second complex is formed by TbEIF4E5, TbEIF4G2, two distinct 14-3-3 homologs (Tb927.11.9530 and Tb927.11.68710, named Tb14-3-3 I and II), and yet another hypothetical protein Tb927.11.6010 (17.9 kDa), this one only found in *Trypanosoma* species [68]. The three hypothetical proteins were originally named according to their predicted molecular weight, however, this nomenclature may cause confusion when comparing orthologs varying in size between different species. Therefore, we opted to rename them here, so that the first protein, Tb927.11.6720, will be called TbG1-IP (TbEIF4G1 *Interacting Protein*) as it binds the TbEIF4E5 complex through TbEIF4G1, while Tb927.11.350 will be named as TbG1-IP2 and Tb927.11.6010 as TbG2-IP.

**Figure 6:**
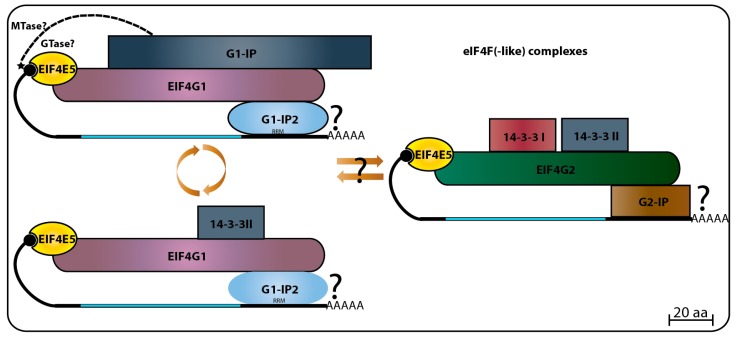
Schematic representation of the eIF4F-like complexes formed by EIF4E5. One eIF4F-like complex performed by EIF4E5 is composed by its interaction with EIF4G1, that binds to the guanylyltransferase/methyltransferase domain-containing protein G1-IP (which seems to be regulated by a 14-3-3 protein homolog) and G1-IP2, an RNA binding protein. The second EIF4E5 complex is composed by its interaction with EIF4G2, which also binds to a protein of unknown function called here G2-IP.

TbG1-IP is a large protein with two distinct domains, guanylyltransferase and methyltransferase, both found in proteins involved in cap addition and modification during mRNA maturation [107], and not yet seen or expected to be present in a cytoplasmic protein involved with translation initiation factors. TbG2-IP is a putative RNA binding protein having two distinct domains associated with RNA binding. Yeast two-hybrid assays have defined that TbG1-IP binds directly to TbEIF4G1, and mass spectrometry assays of the TbG1-IP purified complex have shown that TbEIF4E5, TbEIF4G1, and TbG1-IP2 interact in the absence of the 14-3-3 homolog [68]. The 14-3-3 proteins are phosphoserine/phosphothreonine-binding proteins that bind to a multitude of functionally diverse signaling factors and regulate protein–protein interactions [108,109]. Its absence from the TbEIF4E5–TbEIF4G1–TbG1-IP–TbG1-IP2 complex might imply a dynamic exchange between two complexes based on the TbEIF4E5–TbEIF4G1 interaction. The first complex would include TbG1-IP in the absence of the 14-3-3 homolog, while the second would have the 14-3-3 protein replacing TbG1-IP [68] (Figure 6).

#### 3.3.3. EIF4E6 Binding Partners

**Figure 7:**
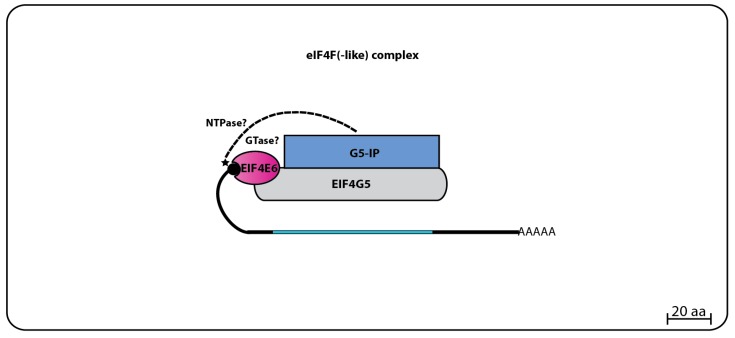
Schematic representation of the eIF4F-like complex of EIF4E6.

The TbEIF4E6 yeast two-hybrid assays revealed a novel direct interaction with the last eIF4G homolog, TbEIF4G5. Again, the interaction was specific, since no other eIF4E homolog bound to TbEIF4G5, while no interaction between TbEIF4E6 and the remaining eIF4Gs was seen. Likewise, mass spectrometry analysis confirmed that TbEIF4E6 associates in vivo with TbEIF4G5, and also forms a novel complex that includes one more hypothetical protein, Tb927.11.14590 (70.3 kDa), that binds directly to TbEIF4G5, named TbG5-IP [96] (Figure 7). Curiously, TbG5-IP also has distinct domains seen in cap forming enzymes, a nucleoside triphosphate (NTP) hydrolase and a guanylyltransferase domain [107]. The presence of proteins with cap generating domains interacting with homologs of translation initiation factors is unprecedented, and raises questions regarding likely functions for the EIF4E5 and EIF4E6 based complexes. Noteworthy is the fact that both eIF4E homologs and the partners having guanylyltransferase domains would be expected to share the same substrate.

#### 3.3.4. EIF4E5 and EIF4E6 Knockdown Effects and Putative Roles

Targeted, RNAi mediated, knockdown experiments have indicated that neither TbEIF4E5 nor TbEIF4E6 are essential for cell viability, cell division, or seem to be required for general translation in procyclic cells [68,96]. These results have been, in part, contradicted by high throughput RNAi assays, showing an abnormal growth effect after TbEIF4E5 knockdown, while TbEIF4E6 depletion led to a significant loss of fitness in procyclic, bloodstream, and differentiating forms [91]. The inability to generate double knockout cell lines for either protein may also be an indication that, in fact, both are essential proteins [68,96]. The targeted TbEIF4E5 knockdown did induce an impairment of cellular motility or swimming, inducing a “settling” phenotype in liquid media [68], a phenomena also observed after knockdown of its putative partners, the 14-3-3 proteins [110]. Related to this, the TbEIF4E5 depletion also interfered with the social motility (SoMo) effect, observed when *T. brucei* cells are grown in semisolid agar plates [68]. In tethering assays, the EIF4E5 partners TbEIF4G1, TbEIF4G2, and TbG2-IP have behaved as activators of mRNA translation or stability [92,93]. In agreement with the similarities in complex formation and binding partners, the TbEIF4E6 RNAi experiments also implied that both TbEIF4E5 and TbEIF4E6 are related in function. Despite the absence of any changes induced by its depletion on apparent motility, and the lack of the “settling” phenotype observed after the TbEIF4E5 RNAi, the TbEIF4E6 knockdown also interfered with the SoMo effect in cells grown in agar plates. It also caused an unusual detachment of the flagellar in cells submitted to mild centrifugation, indicating an impact on the flagellar attachment strength [96]. Overall, the results described so far for TbEIF4E5 and TbEIF4E6 indicate that both are indeed required for efficient cell growth, and that they may act upon the regulation of the metabolism of specific mRNAs, perhaps as part of one or more regulons. These would likely be associated somehow with cellular motility and/or flagellar attachment.

## 4. Concluding Remarks

In this review, the basic properties and the knowledge generated during the last two decades on the six eIF4Es, the cytoplasmic cap binding proteins, from trypanosomatids, has been reviewed with the focus on evaluating possible roles during translation initiation or its control. The uniqueness of the trypanosomatid system can be highlighted by the identification of multiple eIF4F-like complexes, having different eIF4E (six) and eIF4G (five) subunits, as well as novel binding partners with roles yet to be properly defined. It seems plausible to speculate that, in the absence of transcriptional control, a system of multiple eIF4F-like complexes has evolved in order to maximize the control of the fate of different classes of mRNA regulons. This system could then play a role in cell cycle, differentiation and adaptation to environmental changes, such as those observed within the parasites’ different hosts. It is possible, then, to suppose that the evolution of the translation initiation apparatus in trypanosomatids would reflect an adaptation to parasitism on vertebrate hosts. This is unlikely, however, since all *Leishmania* and *Trypanosoma* eIF4Es and partner proteins are found in monoxenous trypanosomatids, which are mostly exclusive to insects, and also in *Phytomonas* species, plant parasites [5]. The multiple eIF4Es and eIF4F-like complexes are unlikely to be related to parasitism, since most of the described trypanosomatid eIF4Es and their partners are found in the related free-living kinetoplastid *Bodo saltans* [2]. Transition from a free-living bacteriovorous lifestyle to parasitic life was followed by significant phenotypic transformations that optimized basic genome functions, re-arranging, and removing redundant (or no longer needed) metabolic pathways [4,111]. In this scenario, the conservation of translation initiation factors during the transition to parasitism in Kinetoplastida denotes an ancient, complex, and unique system that may be required for the control of gene expression in organisms lacking most known mechanisms regulating mRNA synthesis. The multitude of eIF4Es and associated complexes, whose functions are still not fully understood, are thus undoubtedly critical elements for the survival of these pathogens that need to be investigated further.

Among all eIF4E homologs, a direct involvement by EIF4E1 and EIF4E2 during translation initiation is unlikely for several reasons. The lack of eIF4G partners for either protein and their absence from a true eIF4F complex rule out a role in general translation. Furthermore, their reduced abundance, especially for EIF4E2, make them unlikely to be present in levels sufficient to support protein synthesis in large scale [69,75]. Although LmEIF4E1 was proposed to be responsible for translation initiation in axenic amastigotes in *Leishmania*, based on data from *L. amazonensis* where LaEIF4E4 was missing [87], no further direct evidence for a role in translation was reported to date, and in *L. infantum*, sufficient levels of LiEIF4E4 were detected in amastigotes [94]. The fact that TbEIF4E1 does not seem to be essential for cell survival in *T. brucei*, with a minor effect only in bloodstream forms after knockdown [75], also suggest other functions. Indeed, the data from tethering experiments indicated a major suppression effect of TbEIF4E1, and its identified partner 4E-IP [92,93] on mRNA translation. Additionally, its partial localization to P-bodies-like granules under normal growth conditions [72] also points to a possible role repressing the translation of selected mRNAs, similar to what has been described for metazoans 4EHP [58,112]. This activity could be enhanced under stress conditions that induce translation shutdown, and relocation of the translation machinery to stress granules. As EIF4E2 shares several features in common with EIF4E1, although it has not yet been shown to be able to repress translation, it may also act as a regulator of translation acting upon selected mRNA populations.

A role for LmEIF4E3 during translation initiation has been previously discarded in *Leishmania*, based on a lack of co-migration with LmEIF4G4 in sucrose gradient and to its presence, without LmEIF4G4, in stress granules. These properties were postulated as indicative of roles in mRNA storage or degradation [95]. However, TbEIF4E3 participation in stress granules was considered minor in starvation stress granules in *T. brucei* [72], and both TbEIF4E3 and TbEIF4E4 and their partners were found in polysomes from bloodstream cells, although TbEIF4E4 was more abundant [99]. That the presence in stress granules cannot be considered a valid argument to rule out a role in translation is highlighted by the fact that human eIF4E1, the prototype cap binding protein required for translation initiation factor, is also found localized to both stress granules and P-bodies [113]. Furthermore, recent work has shown that almost all *T. brucei* eIF4E and eIF4G homologs were identified as part of stress granules during starvation [114]. TbEIF4E1 seems more suitable as a translation repressor [93], since it is localized to stress granules even under optimal growth conditions, and has been shown to co-localize with DHH1 [72], an ATP-dependent RNA helicase involved in mRNA turnover and decapping [115]. Another argument against a role for EIF4E3 during translation initiation is the fact that LmEIF4E3 does not bind efficiently to the m^7^GTP or cap4 structures [79]. This is also debatable, since the data was produced through in vitro studies and may not reflect what is happens in vivo, especially considering that TbEIF4E3 can be found bound to polysomal mRNAs [93]. Perhaps additional conformation changes are necessary to enhance EIF4E3 affinity to mRNAs in vivo, or it may be required to be part of a complex, perhaps with EIF4G4, to somehow compensate its lower affinity for the cap structure. Nevertheless, its presence in levels far above those observed for its partner EIF4G4, in contrast to EIF4E4 and EIF4G3 which are found with similar abundances in *T. brucei* at least [75,88], does suggest roles other than as part of an eIF4F-like complex. More data are needed to explain the questions raised.

The scenario created by the available data indicates two major complexes, based on EIF4E3 and EIF4E4, performing eIF4F related functions during translation initiation in trypanosomatids. Although all analyzed eIF4E from *Leishmania* and *Trypanosoma* are significantly less abundant than yeast eIF4E, their levels might be adequate within a scenario of multiple active eIF4F complexes and in the absence of a 4E-BP like protein, such as the yeast p20 [75,116]. EIF4E3 and EIF4E4 are the most abundant of the trypanosomatid eIF4Es and even though EIF4E3 is the only homologue confirmed to be essential in both insect and vertebrate life stages in *T. brucei* [75], recent data from *Leishmania* EIF4E4 also confirms its requirement for cell survival [94]. In addition, the fact that both proteins are present in polysomes and are active stimulators of translation is a strong indication for a involvement in general translation initiation [92,93,99]. Significant properties shared in common by both EIF4E3 and EIF4E4, such as their structure, ability to bind to related eIF4G homologs and subcellular localization reinforce the possibility that both can perform bona fide eIF4E roles. Specific features, however, may indicate different requirements regarding mRNA recognition, leading to the binding to different mRNA targets.

The third set of trypanosomatid eIF4Es, containing EIF4E5 and EIF4E6, is characterized by unusual eIF4F-like complexes, which associate eIF4E and eIF4G homologs with proteins having domains found in enzymes responsible for mRNA cap addition and methylation [107]. Although neither EIF4E5 nor EIF4E6 are likely to play roles in general translation, mainly due to their low abundance and to the fact that their knockdown did not affect translation rates, they might be required to regulate translation of specific mRNA subsets. Their absence then would trigger the impairment in cell motility and structural abnormalities [68,96]. A major aspect is their involvement with putative cap-generating proteins. During the cap4 synthesis in trypanosomatids, it is known that the enzyme TbCgm1 is responsible to add the cap0 structure to the 5′ end of the precursor SL RNA [117,118]. Subsequent 2′-*O*-methylations are performed by the methyltransferases TbMTr1 and TbMTr2 for the first two nucleotides, while the third and fourth methylations are made by TbMTr3 [119,120]. All four enzymes localize to the nucleoplasm, compatible with their activities targeting their newly transcribed SL RNA substrate prior to the nuclear *trans*-splicing event. TbG1-IP is related to TbCgm1 in that both have guanylyltransferase and methyltransferase domains[96], although the cytoplasm association of TbG1-IP with the TbEIF4E5–TbEIF4G1 complex would rule out any role targeting the precursor SL RNA. Furthermore, a novel cytoplasmic capping activity has been recently described associated with the enzyme TbCe1. This has been proposed to be part of a novel pathway targeting mRNAs that have undergone decapping in the cytoplasm, but, upon selective recapping by TbCe1, can be reactivated and restored to the pool of translatable mRNAs [121]. Remarkably, TbCe1 is similar in structure to TbG5-IP, part of the TbEIF4E6–TbEIF4G5 complex [96]. It is possible, then, that both EIF4E5 and EIF4E6 based complexes could be part of novel pathways regulating mRNA translation through selective decapping and recapping within the cytoplasm.

**Figure 8:**
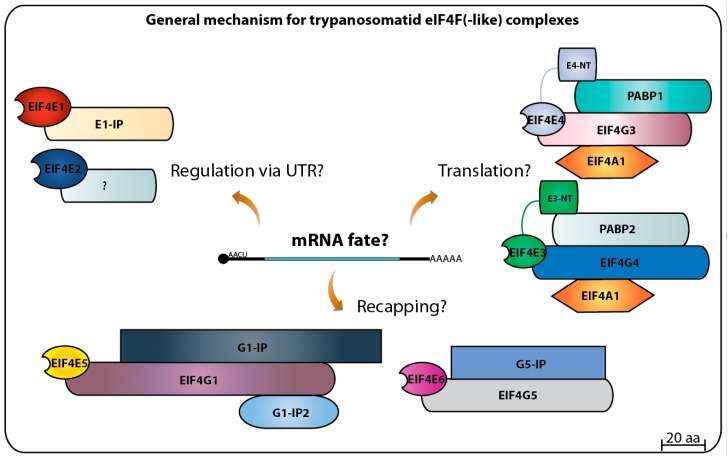
Summary of the eIF4F(-like) complexes formed by the six EIF4E of trypanosomatids. A dynamic picture of multiple eIF4E homologs acting as part of different eIF4F complexes that might regulate the mRNA fate on different aspects of translation initiation.

Altogether, the progress achieved on the study of the eIF4E initiation factors and translation in general in trypanosomatids has raised more questions than it has solved. So far, the picture that emerges is a dynamic one with the multiple homologs acting as part of different complexes targeting multiple steps required for translation to proceed efficiently (summarized in Figure 8). A complex selection process may determine which mRNA associates with which complex. This is likely to require the participation of many of the multitude of RNA binding proteins and others which have relevant roles as modulators of trypanosomatid gene expression [122,123,124]. Some important questions remain to be answered, such as what are the targets recognized by each eIF4F(-like) complex [9], which if any of these complexes play roles during cell differentiation and how their activities are regulated? The last question may also help clarify what aspects are common, and which are unique to the distinct life cycle of parasitic trypanosomatids [125,126]. The full picture of the translation initiation system in trypanosomatids seems to still be far way from being understood. Hence, future approaches are needed to elucidate these questions and others in order to define specific roles for each eIF4E homolog in mRNA metabolism as well as translation, contributing to the understanding of the biology of these impressive organisms that seem to be specialized in doing things their own way.
